# Prospects of Cationic Carbosilane Dendronized Gold Nanoparticles as Non-viral Vectors for Delivery of Anticancer siRNAs siBCL-xL and siMCL-1

**DOI:** 10.3390/pharmaceutics13101549

**Published:** 2021-09-24

**Authors:** Viktar Abashkin, Elżbieta Pędziwiatr-Werbicka, Rafael Gómez, Francisco Javier de la Mata, Volha Dzmitruk, Dzmitry Shcharbin, Maria Bryszewska

**Affiliations:** 1 Institute of Biophysics and Cell Engineering of NASB, 27 Akademicheskaya St., 220072 Minsk, Belarus; shcharbin@gmail.com; 2 Department of General Biophysics, Faculty of Biology and Environmental Protection, University of Lodz, 141/143 Pomorska St., 90-236 Lodz, Poland; elzbieta.pedziwiatr@biol.uni.lodz.pl (E.P.-W.); maria.bryszewska@biol.uni.lodz.pl (M.B.); 3 Department of Organic and Inorganic Chemistry, Research Institute in Chemistry “Andrés M. del Río” (IQAR), Universidad de Alcalá, 28871 Alcalá de Henares, Spain; rafael.gomez@uah.es (R.G.); javier.delamata@uah.es (F.J.d.l.M.); 4 Institute “Ramón y Cajal” for Health Research (IRYCIS), 28034 Madrid, Spain; 5 Networking Research Center on Bioengineering, Biomaterials and Nanomedicine (CIBER-BBN), 28029 Madrid, Spain; 6 Center of Molecular Structure, Institute of Biotechnology CAS, 595 Průmyslová St., 252 50 Vestec, Czech Republic; volha.dzmitruk@ibt.cas.cz

**Keywords:** gold nanoparticles, carbosilane dendrons, siRNA, gene therapy

## Abstract

Cancer is one of the most important problems of modern medicine. At the present time, gene therapy has been developed against cancer, which includes the delivery of anticancer small interfering RNAs (siRNAs) directed at cancer proteins. The prospect of creating drugs based on RNA interference implies the use of delivery systems. Metal nanoparticles are the most studied objects for medicine, including their application as non-viral vectors. We have synthesized gold nanoparticles (AuNPs) modified with cationic carbosilane dendrons of 1–3 generations, with a positive charge on the surface, gold nanoparticles can effectively bind small interfering RNAs. Using a photometric viability test and flow cytometry, we assessed the ability of dendronized gold nanoparticles in delivering siRNAs to tumor cells. The efficiency of the complexes in initiating apoptosis was measured and, also, the overall effect of proapoptotic siRNA on cells. AuNP15 has both the highest efficacy and toxicity. The delivery efficiency in suspension cell lines was 50–60%. Complexes with targeted siRNA decreased cell viability by 20% compared to control and initiated apoptosis.

## 1. Introduction

Oncological disorders remain one of the main problems in medicine. One of many problems, can be the resistance of some types of cancer to chemotherapy and radiation therapy. Against this backdrop, researchers continue to search for effective solutions for cancer therapy. One possible approach could be the use of short-chain non-coding RNAs, namely microRNAs (miRNAs) and siRNAs [1,2]. SiRNAs are short (20–25 base pairs) double-stranded nucleotides that silence genes due to specific binding to matrix RNA, and as a result block the synthesis of target proteins [3,4]. In the context of cancer, normal cells that are transformed begin to proliferate uncontrollably, avoiding apoptosis under the required conditions [5]. A significant family among these proteins is bcl2, normally, this protein regulates mitochondrial activity, promotes the release of cytochrome C and the formation of reactive oxygen species (ROS). It is assumed that the mutation of bcl2 could be one of the causes of both tumorigenesis and other disorders. In addition, mutation could reduce the efficacy of cancer therapy [6,7,8].

Thus, it is possible to initiate apoptosis in cancer cells by silencing the genes of these proteins using siRNA. Due to the absence of a direct effect on intranuclear DNA, siRNAs do not lead to changes in the host cell genome which is a much safer approach to gene therapy when compared to the action of DNA plasmids [9]. One of the main limitations in using siRNAs as therapeutic agents is their inability to enter cells through the cell membrane, along with their destruction by enzymes in the bloodstream [9,10]. This necessitates the use of specific delivery systems. Carriers representing nanostructures of several natures are now very promising: polymers, liposomes, dendrimers, quantum dots, and metal nanoparticles [11]. Earlier, both our group [12,13,14] and other researchers [15,16,17] have shown a fairly high efficiency of dendrimers and other hyperbranched polymers as carriers of siRNAs and other therapeutic agents and gold nanoparticles being attractive. Due to their unique properties, AuNPs have been studied as agents in biomedical applications for several decades [18,19]. In particular, the high compatibility of gold nanoparticles with biomolecules can be useful in gene therapy, in delivering genetic material to target cells [20,21]. In turn, different super-branched macromolecules are also of considerable interest in biology and medicine. Cationic macromolecules are often investigated as vectors of nucleic acids because they form complexes through electrostatic interactions, which aids nuclease protection and delivery to target cells [12,22,23]. However, gold nanoparticles also open up the prospect of multifunctional treatment, e.g., it could be possible to combine siRNA delivery and photothermal therapy.

Functionalization of nanoparticles is an important aspect in reducing their toxic effects, increasing the efficiency of delivery (e.g., accumulation in target tissues) and increasing stability [24,25]. We have previously combined two types of systems by modifying gold nanoparticles with cationic carbosilane dendrons [26,27]. Cationic carbosilane dendrons containing a thiol moiety at the focal point to create good anchorage ligands that help stabilize nanoparticles and simplify synthesis. Nanoparticles combine the properties of both "hard" (metal core) and "soft" (surface dendrons) nanoparticles, which can lead to unexpected properties. "Hard" nanoparticles might accumulate more efficiently both in cells by uptake and at tumor sites due to enhanced permeability and retention (EPR) effect [28,29]. At the same time, the "soft" component of nanoparticles has a greater mobility of branches and less rigidity and, as a result, is better able to bind genetic material due to more efficient molecular packing [30].

In this article, we analyze the levels of uptake of gold nanoparticles and anticancer siRNA complexes on three cell lines, and the viability of the cells after treatment.

## 2. Materials and Methods

### 2.1. Small Interfering RNA

siRNA sequences (Table 1) were synthesized at the Institute of Physical Organic Chemistry of NASB (IPOC NASB) according to known methods. Lyophilized siRNAs were dissolved in a siRNA buffer. The composition of the siRNA buffer is 60 mM KCl (Fisher Scientific UK Ltd., Loughborough, UK), 6 mM HEPES-pH 7.5 (MilliporeSigma, Burlington, MA, USA), and 0.2 mM MgCl_2_ (MilliporeSigma, Burlington, MA, USA). Non-fluorescent siRNAs were used for cytotoxicity assay; siRNAs labeled with FAM (6-Carboxyfluorescein, IPOC NASB, Minsk, Belarus) at the 5′-end of the sense sequence were used in flow cytometric experiments. RndRNA is a random RNA sequence used as control versus targeted siRNA.

### 2.2. Gold Nanoparticles

**Figure 1:**
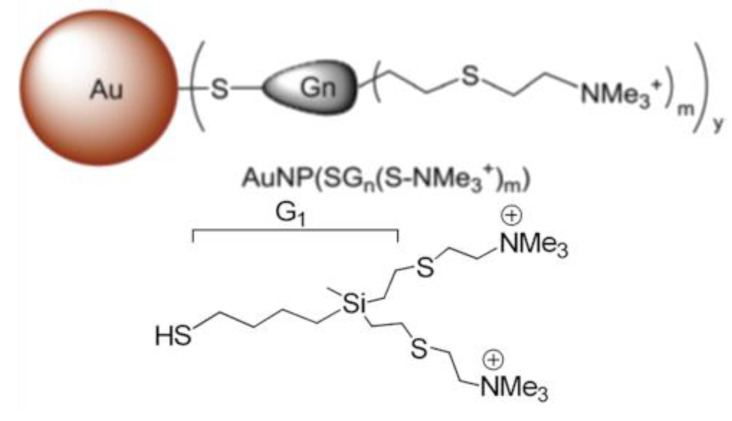
Structures of AuNPs. AuNP13, n = 1, m = 2; AuNP14, n = 2, m = 4; AuNP15, n = 3, m = 8. G_n_ is dendron generation.

Synthesis and characteristics of dendronized gold nanoparticles with cationic carbosilane dendrons (Figure 1, Table 2) have previously been described [26].

### 2.3. Cell Line Culture

Experiments were carried out on HeLa, human acute promyelocytic leukemia (HL-60), and T-cell acute lymphoblastic leukemia (CEM-SS). HeLa cells were grown in full DMEM with stable glutamine, 4.5 g/L glucose (Life Technologies, Paisley, UK), 10% FBS (Life Technologies, Paisley, UK), 100 U/mL penicillin, and 0.1 mg/mL streptomycin (MilliporeSigma, Burlington, MA, USA) at 37 °C in humidified air atmosphere with 5% CO_2_. The HL-60 and CEM-SS cells were cultured in full RPMI-1640 with stable glutamine (Life Technologies, Paisley, UK), 10% FBS (Life Technologies, Paisley, UK), 100 U/mL penicillin, and 0.1 mg/mL streptomycin (MilliporeSigma, Burlington, MA, USA) under the same conditions as HeLa.

### 2.4. Cellular Uptake

The cell lines were seeded at 2 × 10^5^ cells/mL in 500 µL suitable culture medium on 24-well plates (1 × 10^5^ cells/well) and preincubated for 24 h at 37 °C in humidified air atmosphere with 5% CO_2_ before treatment. Complexes of FAM-labeled siRNA and AuNP were added to the cells. Complexes for treatment were incubated for 15 min in PBS using concentrations required for the delivery of 100 nM siRNA-FAM. The cells were incubated for 3 or 24 h and washed in PBS according to the standard procedure.

Samples were analyzed by flow cytometry (CytoFLEX, Beckman Coulter, Indianapolis, IN, USA): 25,000 events with the exclusion of necrotic (by 7-AAD; the number of necrotic cells <1%) and aggregated (SSC-H/SSC-A gate) cells.

### 2.5. Cytotoxicity Studies

The cell lines were seeded at 1 × 10^5^ cells/mL in 100 µL suitable culture medium on 96-well plates (1 × 10^4^ cells/well). They were preincubated for 24 h and a further 72 h after adding complexes. Complexes for treatment were incubated for 15 min in PBS using concentrations needed for the delivery of 100 nM of siRNA.

Cytotoxicity was evaluated by Alamar Blue (Invitrogen™; Life Technologies, Paisley, UK). assay for HL-60 and CEM-SS or MTT-test (Carl Roth, Karlsruhe, Germany) for HeLa. Fluorescence/absorbance measurements used a Wallac 1420 Multilabel Counter (Wallac Oy PerkinElmer, Turku, Finland) at λ_ex_ = 530 nm and λ_em_ = 590 nm for Alamar Blue and λ_abs_ = 570 nm, with using a reference wavelength of 630 nm for MTT. Viability of cells (relative units—r.u.) was calculated relatively to the control (not treated).

### 2.6. Apoptosis-necrosis Test

Cells were seeded at 2 × 10^5^ cells/mL in 500 µL suitable culture medium on 24-well plates (1 × 10^5^ cells/well) and preincubated for 24 h before treatment. FAM-labeled siRNA and AuNP complexes were incubated for 15 min in PBS (PBS tablets, Fisher Scientific UK Ltd., Loughborough, UK) using concentrations needed for the delivery of 100 nM of siRNA-FAM and then added to the cells. The cells were incubated for 48 h and washed twice in PBS. The samples were stained with Annexin V-eFluor™ 450 and 7AAD (Annexin V Apoptosis Detection Kit eFluor™ 450; Invitrogen™; Life Technologies, Paisley, UK).

Samples were analyzed by flow cytometry (CytoFLEX, Beckman Coulter, Indianapolis, IN, USA): 50,000 events were analyzed with the exclusion of aggregated cells (SSC-H/SSC-A gate). Three independent channels (PB, FITC, PerCP) were used to assess apoptosis/necrosis both in the general population and the cells that had accumulated complexes.

## 3. Results

### 3.1. Nanoparticle’s Complexes Cellular Uptake

**Figure 2:**
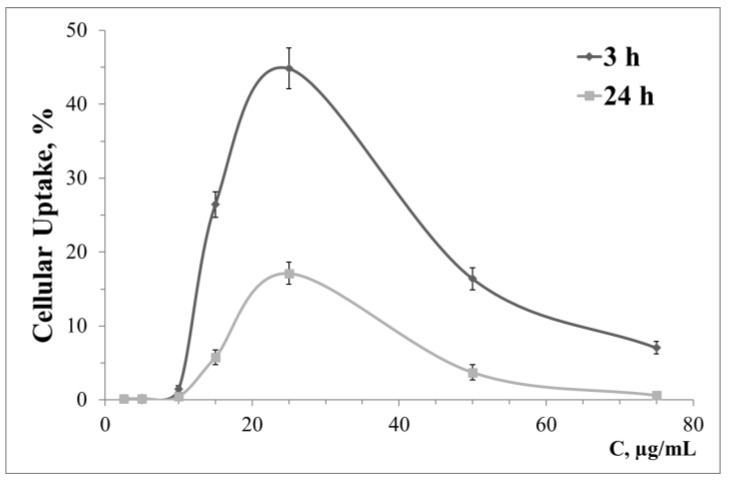
Cellular uptake of complexes with AuNP14 and siRNA (rndRNA-FAM, 100 nM) in HeLa cells after 3 and 24 h incubation. Data obtained based on fluorescence intensity from FAM-labeled RNA by flow cytometry.

The pattern of cellular uptake varies greatly, both with the cell lines used and for the generation of surface dendrons. Figure 2 shows as an example the dependence of HeLa cell absorption of complexes AuNP14-rndRNA on the concentration of AuNP14. Data for other lines and AuNP are given in the Supplementary Materials. Figures S1 and S2 show the dependence of cellular uptake by HeLa on concentration for AuNP13 and AuNP15 nanoparticles, respectively. Cellular uptake of AuNP13, AuNP14, AuNP15 is shown correspondingly in Figures S3–S5 for the HL-60, Figures S6–S8 cell line for the CEM-SS cell line. At low concentrations, AuNP13 did not penetrate the HL-60 and CEM-SS cells. Significantly different signals from the threshold were observed at concentrations of 75 μg/mL and higher. Complexes with AuNP14 had a low level of cellular uptake after 3 h incubation with the same cells. This level increased significantly after one day of incubation. Accumulation of AuNP13-rndRNA and AuNP14-rndRNA complexes in HeLa cells decreased after 24 h incubation compared to 3 h. With AuNP15, cellular uptake did not change with incubation time over the entire concentration range. Uptake of AuNP15 decreased after 24 h incubation compared to 3 h in HL-60 and CEM-SS cells.

**Figure 3:**
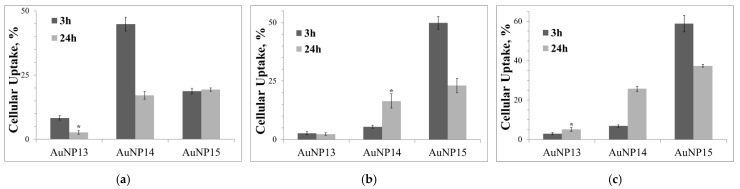
Cellular uptake of complexes with optimal concentrations of AuNP after 3 and 24 hours of incubation in (**a**) HeLa, (**b**) HL-60, and (**c**) CEM-SS. Cells were treated with 100 µg/mL for AuNP13; 25 µg/mL for AuNP14; 10 µg/mL for AuNP15. Used siRNA is rndRNA-FAM (100 nM). Data obtained based on fluorescence intensity from FAM-labeled RNA by flow cytometry. Statistical significance between 3 and 24 h (at * *p* < 0.001) was estimated by the post-hoc Newman–Keuls test.

The optimal delivery concentrations of 100 nM siRNA were 100 μg/mL for AuNP13, 25 μg/mL for AuNP14, and 10 μg/mL for AuNP15. These did not depend on the cell line and were subsequently used in all subsequent experiments. Figure 3 gives a summary of the results for the optima cellular uptake. AuNP13 had a low efficiency in all cases, the maximum uptake being 8.7 ± 0.9% for HeLa cells after 3 h incubation. For the other two cell lines, the same indicator did not exceed 3%. AuNP14 proved to be quite effective with HeLa after 3 h incubation (44.9 ± 2.8%). Uptake in this case decreased by >2.5 times after one day incubation, which might have been due to the mechanisms of utilization by the cells. The complexes did not have time to leave the endosomes and were lysed in them. In its turn, uptake of AuNP14 complexes was higher for 24 h compared to 3 h incubation for HL-60 and CEM-SS cells. However, this is lower than for HeLa cells and amounts to 16.5 ± 3.1% and 25.7 ± 1.3% for HL-60 and CEM-SS cells, respectively. Uptake of AuNP15 complexes did not change over time and reached to ~20% for HeLa cells. AuNP15 complexes also efficiently delivered siRNA to HL-60 and CEM-SS cells; in their case, uptake levels also decreased over 24 h compared to 3 h incubation (49.9 ± 2.6% and 23.1 ± 3.0% for 3 and 24 h incubation, respectively, for HL-60; 58.8 ± 4.2% and 37.4 ± 0.7%, respectively, for CEM-SS).

### 3.2. Cytotoxicity Effects of Dendronized AuNPs and Their Complexes with siRNA

**Figure 4:**
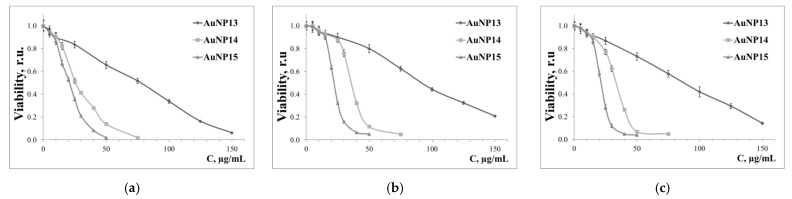
Dose-response curves of (**a**) HeLa, (**b**) HL-60, and (**c**) CEM-SS after 72 h incubation in AuNP. Data obtained from MTT assay normalized to control (untreated) cells.

The cytotoxicity of our nanoparticles was tested after 72 h incubation (Figure 4), which shows that cytotoxicity became more significant with increasing generation of surface dendrons. The most susceptible cell culture was HeLa (IC50 data are in Table 3). HL-60 and CEM-SS cells were more resistant to the effects of AuNP. The parameters of the dose-response curve were also identical for these cell lines. The previously selected concentrations for AuNP15 had only low toxicity to HL-60 and CEM-SS cell lines, with viabilities was 94.5 ± 2.4% and 93.3 ± 3.1% for HL-60 and CEM-SS cells, respectively.

**Figure 5:**
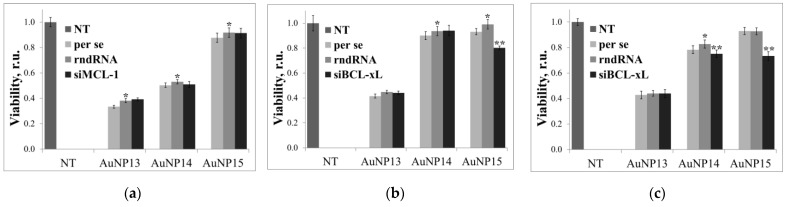
Cytotoxic effect of AuNPs per se and their complexes with target siRNA (siMCL-1 or siBCL-xL and RNA with a random sequence (rndRNA) on (**a**) HeLa, (**b**) HL-60, and (**c**) CEM-SS cells after 72 h incubation. AuNP concentrations correspond to optimal delivery concentrations based on previous calculations (100 µg/mL for AuNP13; 25 µg/mL for AuNP14; 10 µg/mL for AuNP15). Data obtained from MTT assay normalized to untreated cells (NT). Statistical significance between AuNP per se and AuNP-rndRNA (* *p* < 0.01); AuNP-rndRNA and AuNP-siRNA (** *p* < 0.005) was estimated by the post-hoc.

Figure 5 shows data on the cytotoxicity of AuNP complexes with target siRNA (siMCL-1 for HeLa cells; siBCL-xL for HL-60 and CEM-SS cells) and a random sequence (rndRNA) as control. The random RNA should not be incorporated into the RNA interference mechanism, therefore showing the toxicity of the complexes.

For HeLa cells, no significant effects were observed. In some cases, the complexes are slightly less toxic than pure AuNPs, which can be caused by size effects; however, the effect is slight and will be excluded from any further consideration. A significant difference between the target and random sequences was found when using AuNP15 for HL-60 and CEM-SS cells. Survival was reduced by ~20% for both lines. Complexes based on AuNP14 were significantly different between sequences only for CEM-SS (82.7 ± 3.2% for rndRNA and 75.2 ± 2.9% for siBCL-xL).

### 3.3. Assessment of Cell Death Due to Exposure to Complexes

In the following, only AuNP14 and AuNP15 effects on the HL-60 and CEM-SS cell lines will be considered since AuNP13 affected the target siRNA in any case, and there was no effect from the use of the target siRNA for HeLa cells.

The types of cell death based on the staining of cells were analyzed with the Annexin V protein (a marker of apoptosis) and the intercalator 7AAD (a marker of necrosis) by flow cytometry. Cells stained with Annexin V alone belong to the early apoptosis fraction (phosphatidylserine is located on the outer side of the membrane; there are no pores in the cell). The cells that fluoresce in the 7AAD channel are cells in an early stage of necrosis (phosphatidylserine has not yet been released to the outer membrane, but there are pores in the lipid bilayer where 7AAD freely passes). Cells stained simultaneously with Annexin V and 7AAD can be assigned to the late stage of apoptosis. Unstained cells are referred to as the population of living cells.

**Figure 6:**
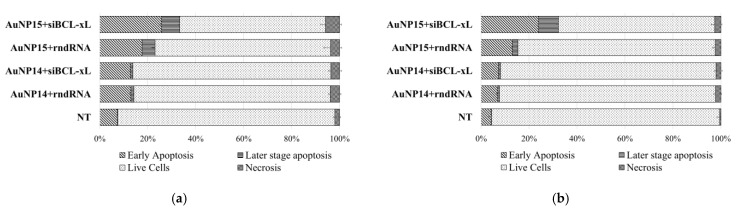
Distribution of (**a**) HL-60 and (**b**) CEM-SS cells by types of cell death among the entire population of cells after 48 h incubation. AuNP concentrations correspond to optimal delivery concentrations based on previous calculations (100 µg/mL for AuNP13; 25 µg/mL for AuNP14; 10 µg/mL for AuNP15). Data obtained by flow cytometry. In each repeat at least 50,000 events were collected and analyzed.

Figure 6 shows the distribution of the cell population by fractions corresponding to apoptotic or necrotic death. The normal level of apoptotic cells in culture should not exceed 10% in the absence of any disturbing influence, and the number of necrotic cells should not exceed 5%. The number of double-stained cells should therefore be vanishingly small. The proportion of cells in early apoptosis in control HL-60 (NT) was 7.4%; in control CEM-SS it was 4.2%. The level of necrosis significantly differed from the threshold value for HL-60 cells, amounting to 2.0 ± 0.3%.

The size of the apoptotic fraction increased upon the addition of AuNP complexes with both the target siRNA and a random sequence. Moreover, there were no differences between complexes based on different siRNAs in the case of AuNP14; the corresponding populations differ to within a standard deviation. In the case of AuNP15, there was a significant difference in apoptosis for the target and random sequences for both cell lines. The level of necrosis in all cases was low, with a maximum value being 5.87 ± 0.75% for the AuNP15-siBCL-xL complexes for HL-60 cells.

**Figure 7:**
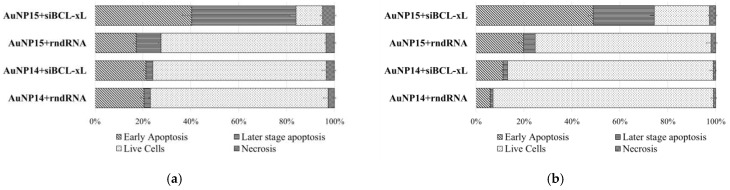
Distribution of (**a**) HL-60 and (**b**) CEM-SS cells by types of cell death among the FAM-positive cells after 48 h incubation. AuNP concentrations correspond to optimal delivery concentrations based on previous calculations (100 µg/mL for AuNP13; 25 µg/mL for AuNP14; 10 µg/mL for AuNP15). Data obtained by flow cytometry. In each repeat at least 7500 events were collected and analyzed.

A more interesting picture was observed when only FAM-positive cells were analyzed, i.e., cells into which the AuNP/FAM-labeled siRNA complexes had internalized (Figure 7). Thus, with AuNP15-siBCL-xL most of the cells within a given population were at early or late stages of apoptosis. The differences between the target and random sequences became more pronounced. Moreover, the effect of the AuNP14-siBCL-xL complexes on the CEM-SS cell line became discernible. This effect was not seen in the general population, but in the population of FAM-positive cells the level of early apoptosis significantly changed from 5.9 ± 1.0% to 11.2 ± 1.1%.

## 4. Discussion

We have investigated the ability of gold nanoparticles modified with cationic carbosilane dendrons of the 1st (AuNP13), 2nd (AuNP14), and 3rd generation (AuNP15) to deliver siRNA to tumor cells. The mechanisms of cell death and general cytotoxic effects caused by these nanoparticles were investigated.

Dendronized gold nanoparticles penetrated cells more efficiently with an increase in the generation of surface dendrons. In the case of AuNP13, the optimal delivery concentrations were quite high and can themselves cause serious toxicity. Cellular uptake was critically small. On the other hand, AuNP14 and AuNP15 proved more toxic, but the optimal concentrations for delivery were much lower. Uptake of complexes with AuNP15 reached 50–60% for HL-60 and CEM-SS cells after 3 h and dropped 1.5–2.5 times after 24 h incubation. In experiments to determine cell death after 2 days of incubation, the uptake level did not drop so much, but remained at 30% for CEM-SS cells and 15% for HL-60 cells. The decrease in uptake was most likely due to both an increase in the number of cells and the degradation of complexes in lysosomes or the removal of complexes from the cell by other defense mechanisms [31,32]. It should be noted that, according to the generally accepted hypothesis, endosomes begin to transform into lysosomes after the uptake of the complexes by endocytosis. A mechanism called the “proton sponge” comes into play. The branches of the dendrons are protonated, their rigidity and, as a result, the volume occupied increases, as a result of which the endosome is destroyed, siRNA released from complexes [33,34]. On this basis, it can be understood why complexes with AuNP15 were most effective; the most likely part of the siRNA leaves the complexes after 24 h and begins to participate in the silencing of genes. The FAM-labeled sense strand is used by the cell at this time, due to which the apparent level of cellular uptake is reduced. The uptake level of AuNP15 did not change either after 3 or 24 h in HeLa cells over the entire studied curve. Since this effect was not found in HL-60 and CEM-SS cells, it can be assumed that there are specific cellular mechanisms that are unable to “clear” the cell from nanoparticles carrying G3 dendrons on the surface, but the mechanism remains unclear.

AuNP14 uptake by the HL-60 and CEM-SS cells line increased after 24 h at optimal delivery concentrations. After 2 days, uptake begins to decline, but AuNP15 complexes successfully accumulate in the cell after 3 h, and then the content of the complexes noticeably decreases. However, the AuNP14 complexes only reach their maximum internalization after one day. This could mean that the ability to enter cells is directly related to the generation of surface dendrons.

Importantly, there can be a complex dependence of the level of cellular uptake on the used concentration of AuNP. For example, the signal from AuNP14 complexes at low concentration is seen after 3 h incubation, but completely disappears after 24 h incubation in HL-60 cells.

We have previously demonstrated [26,27] that gold nanoparticles successfully also bind siRNA. AgNP-siRNA complexation was characterized using confocal microscopy, TEM, agarose gel electrophoresis, zeta potential, dynamic light scattering, and circular dichroism. Summarized data for zeta potential and size are shown in the Table 4. In previous works with dendrimers and dendrimer-like structures [13,35,36,37] the most efficient delivery is carried out with a 10-fold excess of the cationic charge over the negative charge of nucleotides. Despite the investigated gold nanoparticles being dendrimer-like structures, they have a higher rigidity due to their metal core. Therefore, we checked whether this rule will be preserved in this case. Analysis of uptake showed that it was dependent on the concentration of gold nanoparticles, which gives a bell-shaped curve. The optimum does not depend on the cell line, but only on the generation of surface dendrons. Optimal delivery concentrations were 100 μg/mL for AuNP13, 25 μg/mL for AuNP14, and 10 μg/mL for AuNP15. In terms of the surface dendron charge, this is ~40:1 for AuNP13, 15:1 for AuNP14 and 7.5:1 for AuNP15. Thus, an excess of the cationic charge is also necessary in this case. However, if in the case of dendrimers ratio did not depend on generation, it existed here. This is easily explained by the presence of a rigid metal core, which makes the nanoparticle less flexible. While dendrimers can change shape markedly interacting with short strands of oligonucleotides, AuNPs retain their shape; the smaller the generation of surface dendrons, the less they can change shape. This means that it will be more difficult for them to tightly bind with nucleic acids, being fixed on a rigid metal core.

The cytotoxicity of AuNP is directly related to superficial dendrons; higher generation of dendrons caused a more significant decrease in viability in all cases. This effect is readily explained by an increase in the density of the cationic charge on the surface of nanoparticles. Despite cationic charges being required for efficient transfer of genetic material through non-viral vectors, some evidence confirms the high cytotoxicity of such systems. These effects occur for a number of reasons, among which are the violation of the integrity of cell membranes and the generation of reactive oxygen species [38,39]. It is also interesting that at low concentration (<20 μg/mL), cytotoxic effects do not depend on the generation of surface dendrons for the three cell lines used. It seems that only the dimensional properties of nanoparticles are relevant.

To reveal the effect of gene silencing, cytotoxicity of AuNP complexes with targeted siRNAs were compared with complexes of short RNAs with a random sequence, similarly modified. A significant change in cytotoxicity after 3 days incubation was seen only for AuNP15 on HL-60 and CEM-SS cells. The changes compared to the control siRNA were ~20%. Complexes based on AuNP14 worked only for CEM-SS cells. Decrease in cell survival in comparison with the AuNP14-rndRNA complexes was 7.5%. With HeLa cells, no effects were seen with siMCL-1. Moreover, in the case of commercial transfectants (data not shown), this siRNA successfully induced cell death of HeLa cells. It can be assumed that there are some mechanisms and biophysical conditions that do not allow the effective release of target siRNA into the cytosol of a certain cell type. Nevertheless, based on our results, AuNP15 complexes can successfully accumulate in cells, which could become promising for other approaches, e.g., photothermal therapy (PTT).

Since we tried to induce tumor cells to die through apoptosis by blocking proteins of the bcl2 family, it was also necessary to analyze the types of cell death. Thus, increased necrosis will indicate that the complexes have damaged cells in one way or another (for example, by breaking through the cell membrane and forming multiple pores), whereas a high level of apoptosis will indirectly indicate the successful participation of siRNA in RNA interference. This method cannot accurately assess the level of necrosis, since the “fragments” of cells obtained as a result of cell destruction are washed away during sample preparation. With this type of imaging, cells in the earliest stages of necrosis could be found, with damaged membranes but still retaining overall structural integrity and shape.

Cell death was analyzed after 48 h, since it takes about a day for a successful release of siRNA from complexes and the triggering of gene silencing [13,40]. It takes some time longer for the cells lacking proteins of the bcl2 family to enter apoptosis. With a shorter incubation time, the level of apoptosis can be quite low. When cells with complexes are incubated for a longer time, those that have undergone apoptosis will already have been destroyed, and the number of cells in which the necessary gene silencing process has not occurred will increase due to proliferation.

Analysis of the distribution of types of death in the general population of cells confirmed the data on cytotoxicity. AuNP15 proved to be the most effective. The proportion of living cells (twice negative fluorescence signal) was 72.8% for AuNP15 + rndRNA complexes, whereas for AuNP15-siBCL-xL the figure is 61.0%. The control value was 90.5% for all registered events. On the basis of the latter hypothesis, it can also be concluded that the AuNP complexes themselves can cause cell death by apoptosis, while the apoptosis index for AuNP15 was higher than for AuNP14. This may be a sign of the influence of carbosilane dendrons on the process. There are reports [41,42] that spherical gold nanoparticles can increase the level of ROS in tumor cells and initiate apoptosis. These effects are associated with disruption of the mitochondrial membrane potential. Thus, in our case, two complementary mechanisms of apoptosis are quite probable, i.e., along the siRNA pathway and from AuNP that have left the complexes inside the cell.

The approach using the analysis of FAM-positive cells is more interesting. Since the FAM-label is located on the siRNA sense strand not involved in RNA interference, this method should not affect the overall gene silencing process. This has been tested and confirmed in a number of screening studies (data not shown) comparing the effect of labeled and unlabeled siRNA, in which no statistically significant differences were found; a three-color panel analysis for these studies was considered fair.

This method can demonstrate that in the population of FAM-positive cells for the AuNP15-siBCL-xL complexes that had penetrated HL-60 cells, the percentage of PB-positive events (Annexin V) was 84.4%, which is three times higher than in cells with AuNP15-rndRNA complexes (27.5%). The corresponding values are 74.4% and 24.6% for CEM-SS cells, respectively. As with the cytotoxicity study, AuNP14 complexes showed some difference only in the case of CEM-SS, but the change is quite small (13.1% and 6.5% PB-positive events for AuNP14-rndRNA and AuNP14-siBCL-xL, respectively). Moreover, this difference was not seen when the entire population was analyzed.

The fraction of necrotic cells both in the general population and the population of FAM-positive cells is approximately equal. This insignificant effect is most likely caused by free nanoparticles detached from the complexes.

The question is raised by the cell fraction, designated as the late stage of apoptosis. It is impossible to say with any degree of certainty which process of death began earlier. A situation could arise that the cell, being already slightly damaged (7AAD freely penetrates), started undergoing apoptosis. On the other hand, the movement of phosphatidylserine is ATP-dependent, and if cell functions are impaired, then phosphatidylserine can successfully migrate to the outer cell membrane, where it can bind to Annexin V. However, these conclusions can be fully valid for complexes carrying RNA with a random sequence. From our data, there is a pronounced difference between the levels of the late stage of apoptosis under the action of the AuNP15-rndRNA and AuNP15-siBCL-xL complexes, which cannot be explained alone by the above-described effects. In this aspect, it can be assumed that the penetration of the complexes through endocytosis thins the cell membrane (part of the lipid bilayer is spent in the formation of endosomes). After the initiation of apoptosis, the membrane is still insufficiently stable and the formation of apoptotic vesicles on the surface of the membrane leads to its rupture.

Similar advances have been observed in other studies investigating modifications of gold nanoparticles. Some authors [43] achieved ~30% cellular uptake by LNCaP cells of AuNPs modified with polyethyleneimine and folic acid. In the same study, the expression level of the silenced gene was reduced by ~20% compared to the controls. Other authors [44] modified AuNP first with PEG, and then siRNA with a biodegradable linker. Some of the compounds almost completely silenced the Luciferase gene in modified HeLa cells. Gold nanoparticles AuNPs modified by branched polyethyleneimine (bPEI) were used to silence the c-Myc gene in HuH-7 cells [45]. Distribution in the cell and a decrease in protein expression also indicated the potential of this modification. AuNPs were used as a core for a more complex structure, among other things, carrying glucose molecules for more efficient penetration of target cells [46]. The nanoparticles had a high cellular uptake due to their modification with glucose. However, similar modifications involve the use of linear polymers. We used branching polymers, which allows more efficient use of the volume above the sphere of the nanoparticle. However, our nanoparticles have no modifications for active targeted delivery. Further modifications with peptides, oligosaccharides, or other components that facilitate the delivery and release of nucleic acids should solve this problem.

## 5. Conclusions

We have investigated the ability of gold nanoparticles modified with cationic carbosilane dendrons to act as vectors for siRNA delivery to tumor cells. These nanoparticles were quite toxic for the three cell lines used. However, optimal concentrations for siRNA delivery did not significantly reduce cell survival. Relatively high efficiency was found using AuNPs with G3-dendrons on the surface against leukemic cell lines; nevertheless, the required effect is small. Further research is needed to find optimal conditions, siRNA sequences, and modifications of surface dendrons. In the future, other important properties of gold nanoparticles need to be explored, e.g., the effect of surface plasmon resonance.

## Figures and Tables

**Table 1:** Sequences of siRNAs.

Name	Sense	Antisense
siMCL-1	5′-GGACUUUUAUACCUGUUAUdTdT ^1^-3′	5′-AUAACAGGUAUAAAAGUCCdTdT-3′
siBCL-xL	5′-AUAACAGGUAUAAAAGUCCdTdT-3′	5′-CUCUGAUAUGCUGUCCCUGdTdT-3′

^1^ dTdT—stabilizing tail of 2 deoxyribonucleotides, partially protecting against RNAse recognition.

**Table 2:** Physicochemical parameters of AuNPs.

AuNP	Chemical Formula	MW, g/mol	Number of Surface Groups	Diameter, nm
AuNP13	Au180(C19H45Cl2N2S3Si)59	64,762	118	1.8
AuNP14	Au329(C41H97Cl4N4S5Si3)132	201,106	528	2.2
AuNP15	Au247(C85H201Cl8N8S9Si7)132	307,483	948	2.0

**Table 3:** Half maximal inhibitory concentration (IC50) for the studied cell lines.

Gold Nanoparticle	HeLa	HL-60	CEM-SS
AuNP13	67.5 µg/mL	90.0 µg/mL	80.9 µg/mL
AuNP14	26.1 µg/mL	35.7 µg/mL	32.4 µg/mL
AuNP15	19.8 µg/mL	22.1 µg/mL	21.1 µg/mL

**Table 4:** Zeta-potential and hydrodynamic size of AuNP-siBCL-xL complexes on plateau [27].

Gold Nanoparticle	Zeta-Potential, mV		Hydrodynamic Size, nm
	siBCL-xL	siMCL-1	siMCL-1
AuNP13	20.76 ± 0.18	22.59 ± 0.88	783 ± 115
AuNP14	24.53 ± 2.33	22.0 ± 0.78	326 ± 60
AuNP15	25.33 ± 2.45	35.87 ± 3.41	703 ± 113

## Data Availability

The data presented in this study are available on request from the corresponding author without any restrictions.

## Supplementary Materials

The following are available online at https://www.mdpi.com/article/10.3390/pharmaceutics13101549/s1, Figures S1–S8: Cellular uptake of complexes with AuNP and siRNA after 3 and 24 h incubation.
