# Supplementary material for: Prospects of Cationic Carbosilane Dendronized Gold Nanoparticles as Non-viral Vectors for Delivery of Anticancer siRNAs siBCL-xL and siMCL-1
Source: Pharmaceutics. 2021 Sep 24;13(10):1549. doi: 10.3390/pharmaceutics13101549 (PMC8540611; doi:10.3390/pharmaceutics13101549)
Supplement: Supplementary file 1 [file pharmaceutics-13-01549-s001.zip › pharmaceutics-1363412-supplementary.pdf]

pharmaceutics

MDPI

Supporting information

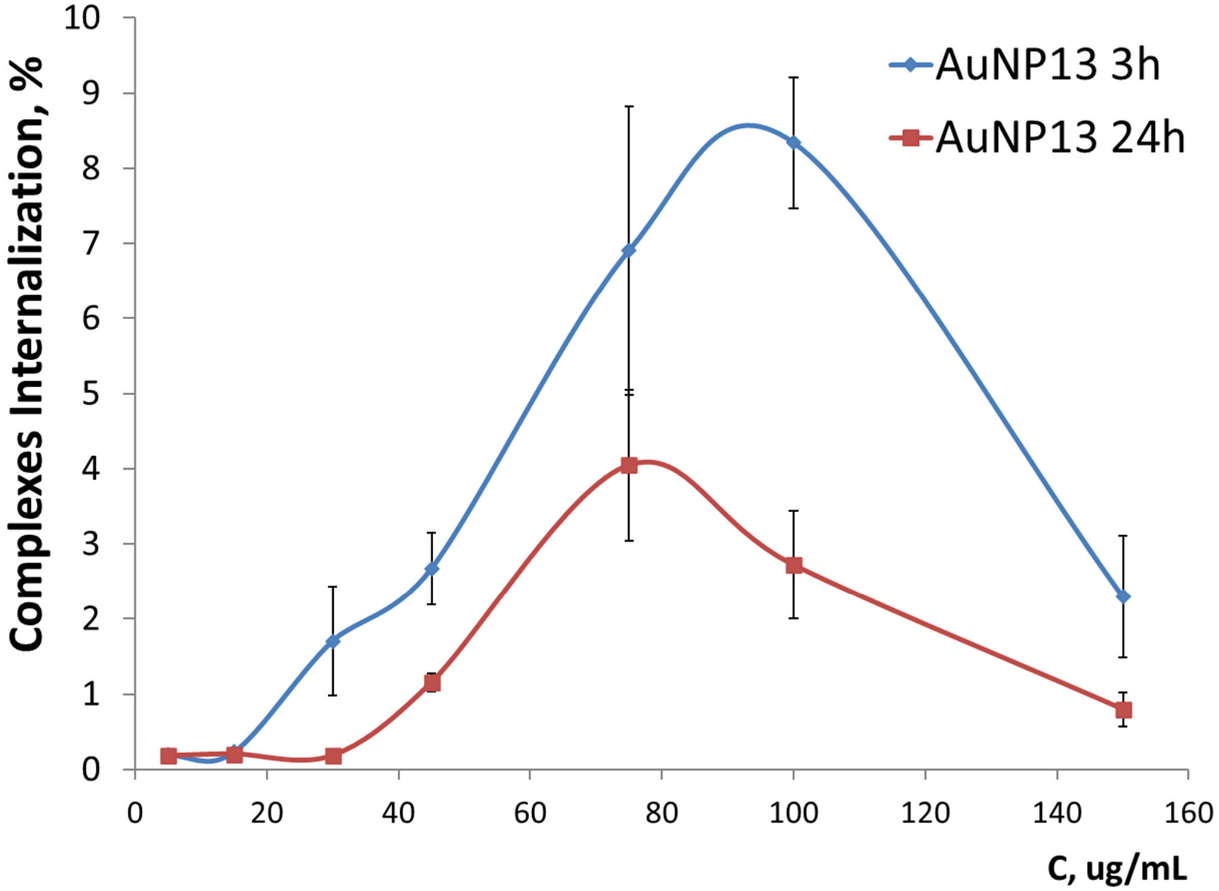

Figure S1. Cellular uptake of complexes with AuNP13 and siRNA (rndRNA-FAM, 100 nM) in HeLa cells after 3 and 24 h incubation. Data obtained based on fluorescence intensity from FAM-labeled RNA by flow cytometry.

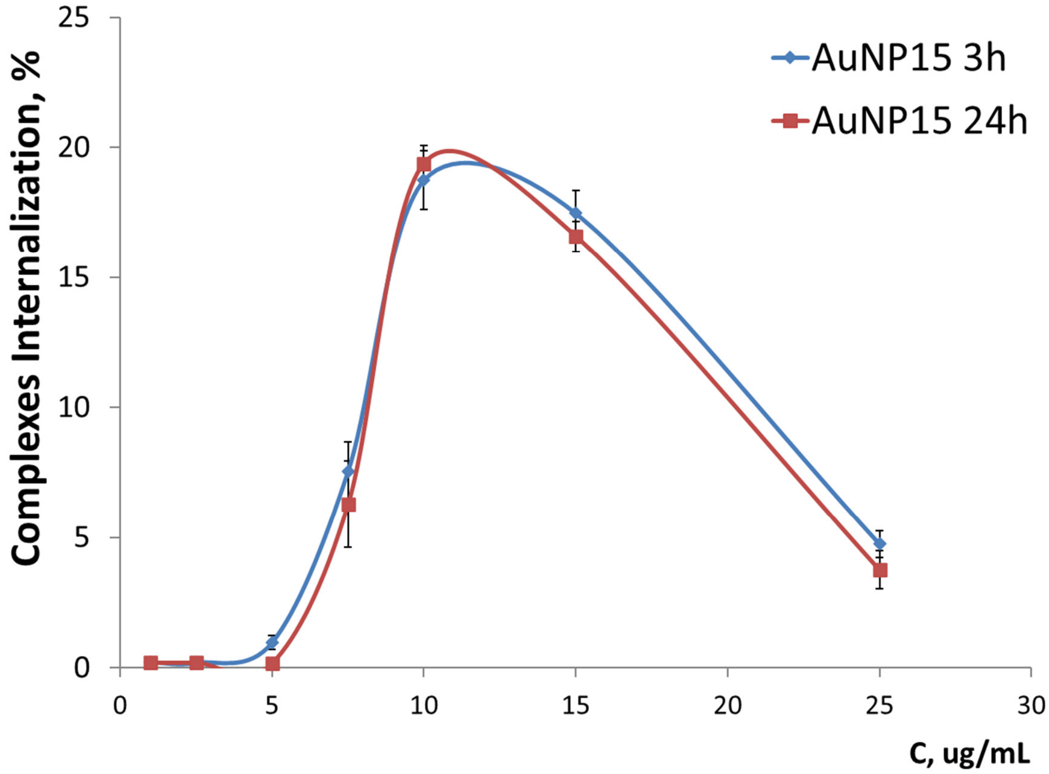

Figure S2. Cellular uptake of complexes with AuNP15 and siRNA (rndRNA-FAM, 100 nM) in HeLa cells after 3 and 24 h incubation. Data obtained based on fluorescence intensity from FAM-labeled RNA by flow cytometry.

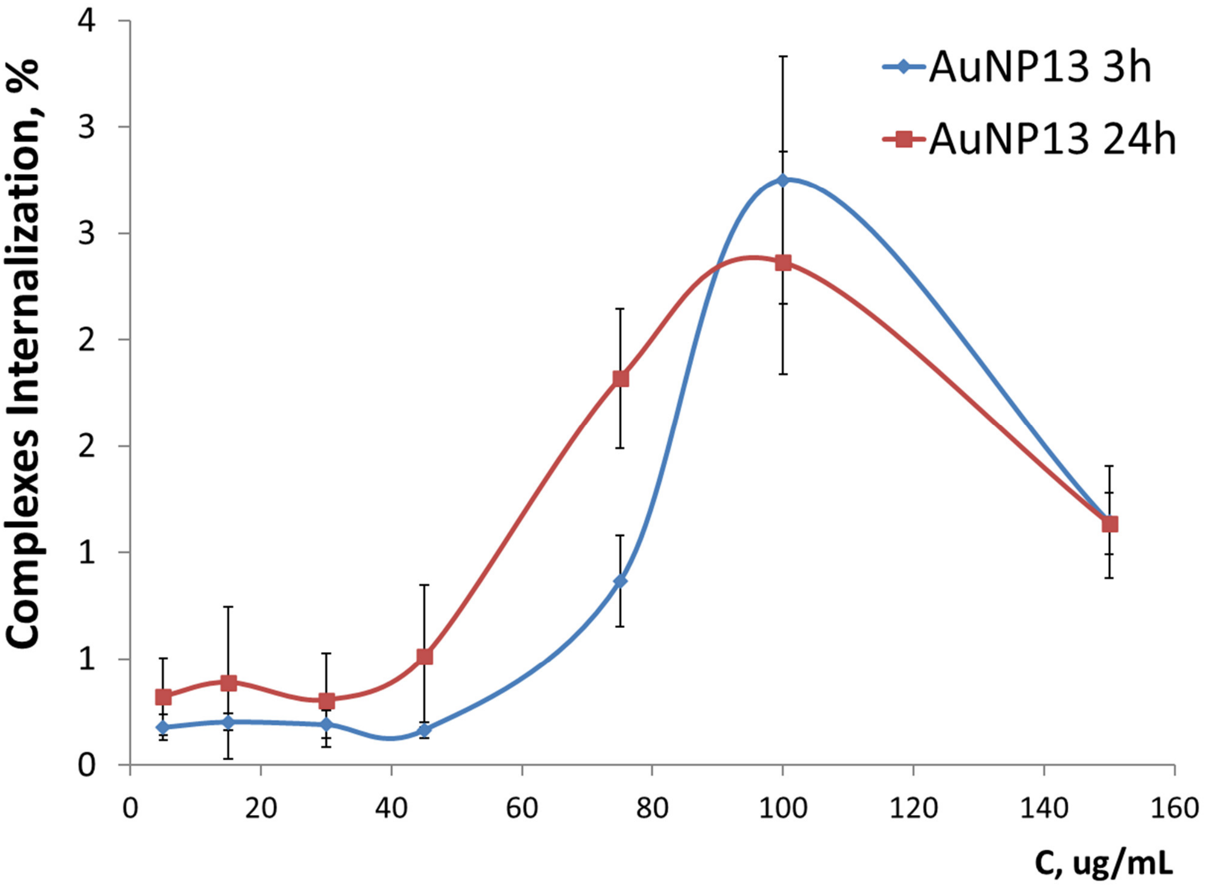

Figure S3. Cellular uptake of complexes with AuNP13 and siRNA (rndRNA-FAM, 100 nM) in HL-60 cells after 3 and 24 h incubation. Data obtained based on fluorescence intensity from FAM-labeled RNA by flow cytometry.

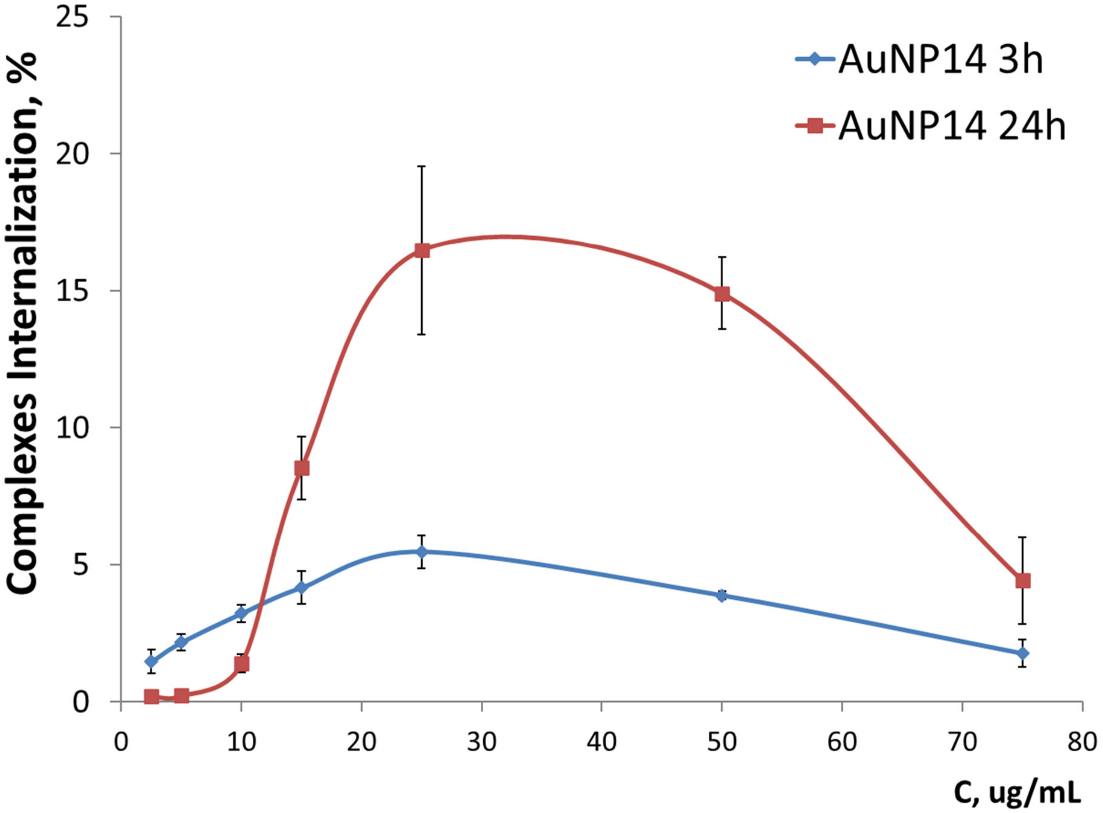

Figure S4. Cellular uptake of complexes with AuNP14 and siRNA (rndRNA-FAM, 100 nM) in HL-60 cells after 3 and 24 h incubation. Data obtained based on fluorescence intensity from FAM-labeled RNA by flow cytometry.

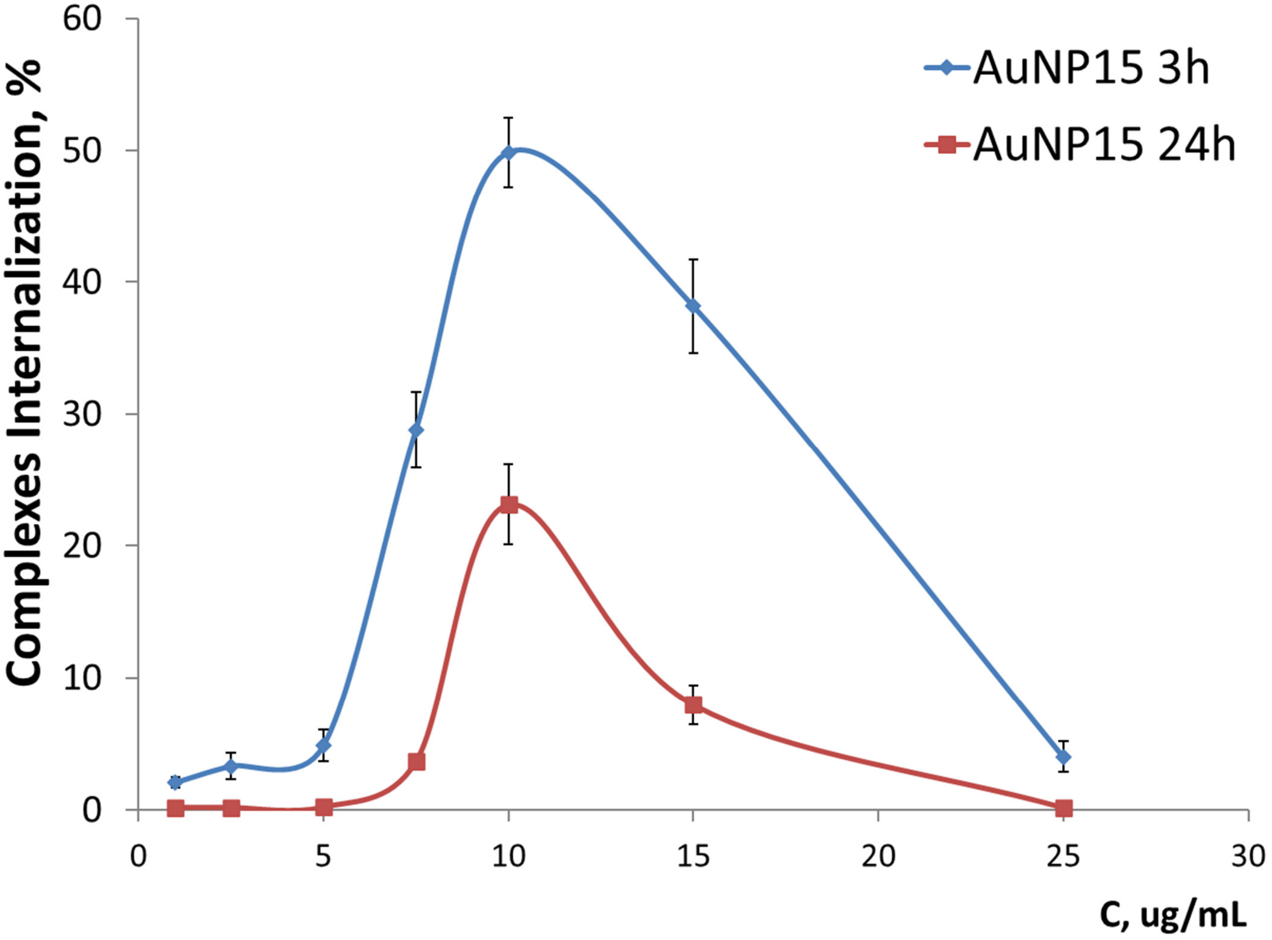

Figure S5. Cellular uptake of complexes with AuNP15 and siRNA (rndRNA-FAM, 100 nM) in HL-60 cells after 3 and 24 h incubation. Data obtained based on fluorescence intensity from FAM-labeled RNA by flow cytometry.

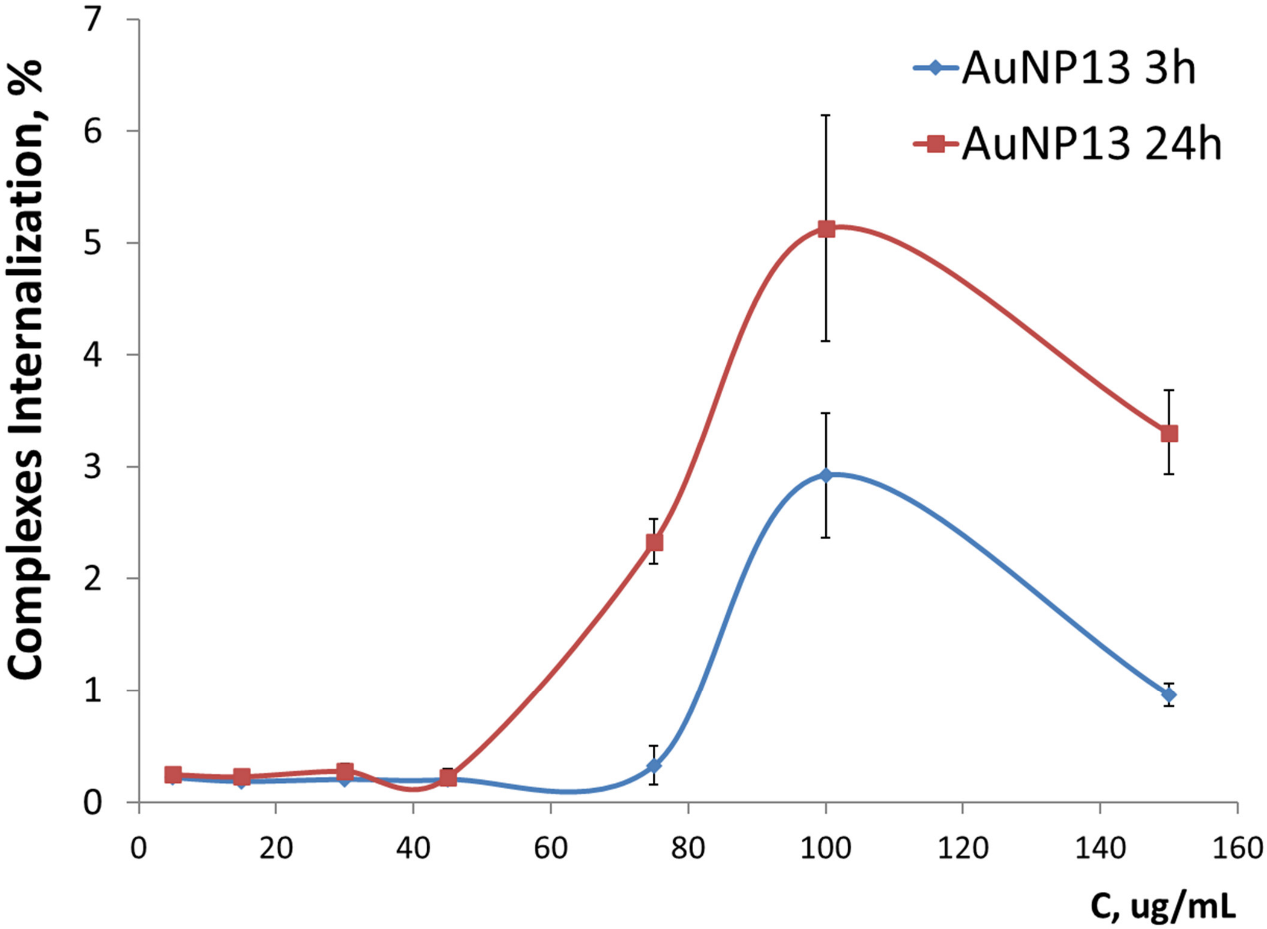

Figure S6. Cellular uptake of complexes with AuNP13 and siRNA (rndRNA-FAM, 100 nM) in CEM-SS cells after 3 and 24 h incubation. Data obtained based on fluorescence intensity from FAM-labeled RNA by flow cytometry.

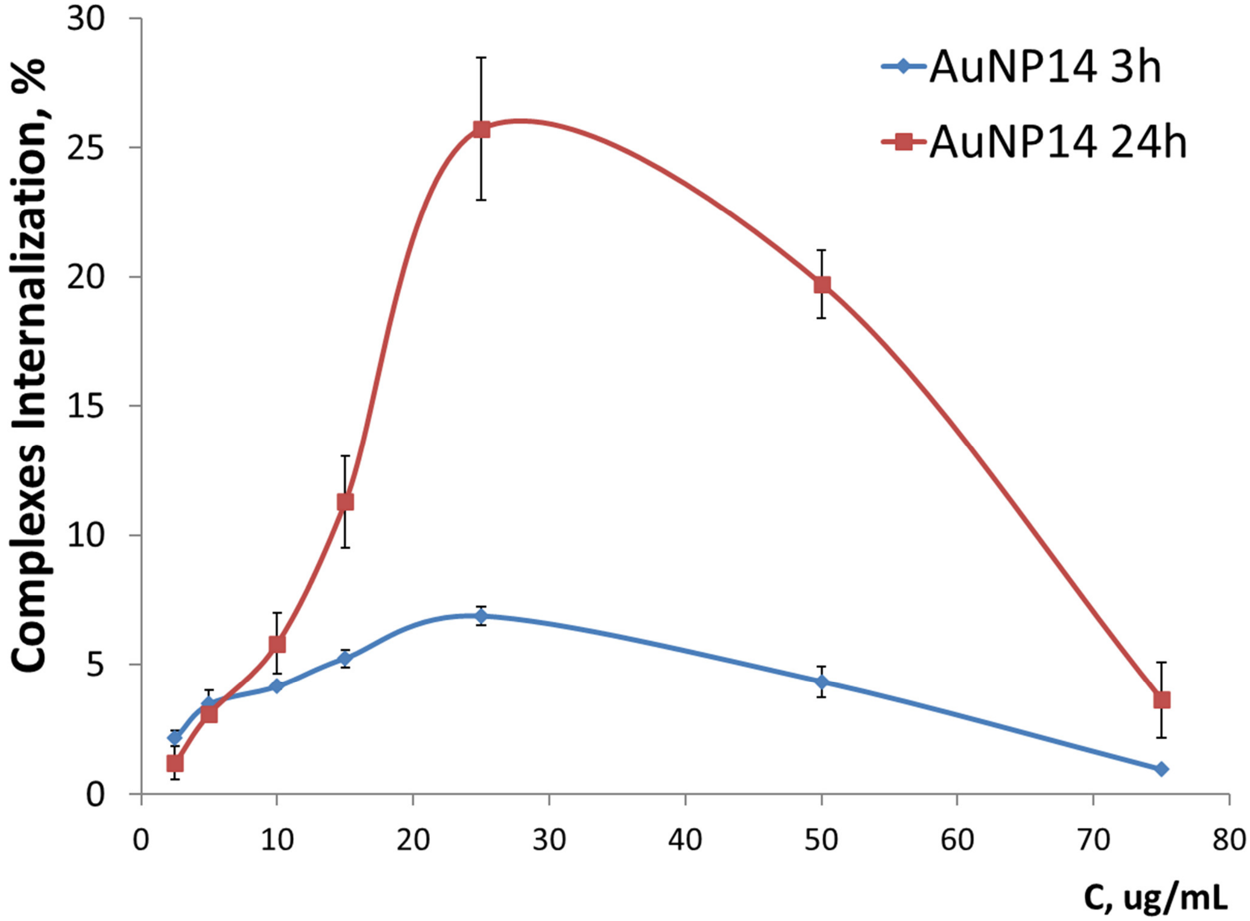

Figure S7. Cellular uptake of complexes with AuNP14 and siRNA (rndRNA-FAM, 100 nM) in CEM-SS cells after 3 and 24 h incubation. Data obtained based on fluorescence intensity from FAM-labeled RNA by flow cytometry.

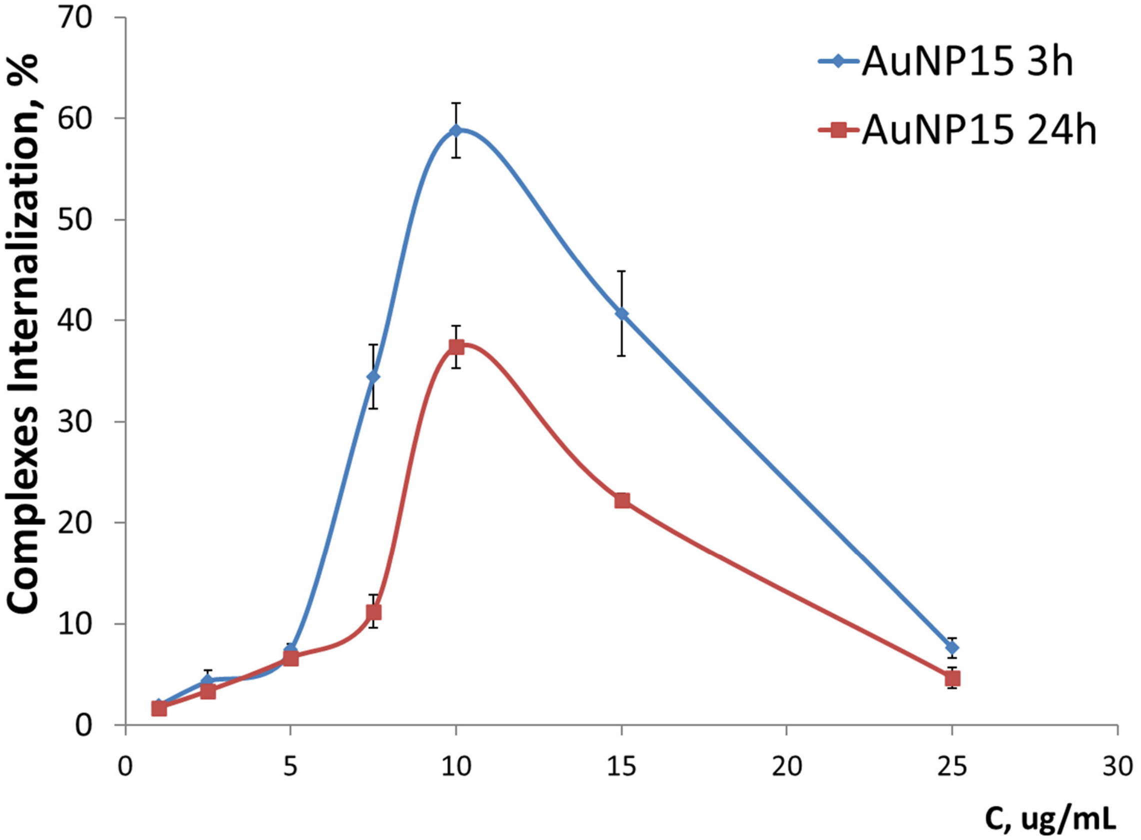

Figure S8. Cellular uptake of complexes with AuNP15 and siRNA (rndRNA-FAM, 100 nM) in CEM-SS cells after 3 and 24 h incubation. Data obtained based on fluorescence intensity from FAM-labeled RNA by flow cytometry.
